# Social Participation: A Strategy to Manage Depression in Disabled Populations

**DOI:** 10.1080/08959420.2023.2255492

**Published:** 2023-09-12

**Authors:** Jiangyun Chen, Li Gan, Yusupujiang Tuersun, Man Xiong, Ju Sun, Chichen Zhang, Haomiao Li

**Affiliations:** a School of Health Management of Southern Medical University, Guangzhou, Guangdong, China; b School of Political Science and Public Administration, Wuhan University, Wuhan, Hubei, China; c ACACIA Labs of SMU Institute for Global Health (SIGH) and Dermatology Hospital, Southern Medical University (SMU), Guangzhou, Guangdong, China; d Institute for Health Management, Southern Medical University, Guangzhou, Guangdong, China

**Keywords:** CES-D, depression, disability, middle-aged and older, social participation

## Abstract

This study aimed to investigate whether social participation (SP) can decrease depressive symptom severity in disabled older adults. A total of 5,937 disabled participants (4877, 1970, 219, and 8 participants responding 1, 2, 3, 4 times, respectively), obtained from the China Health and Retirement Longitudinal Study, were enrolled in our analysis. Based on pooled Ordinary Least Square regressions, SP was associated with decreased depressive symptom severity, and this association was significant in recreational activities and interacting with friends. For brain-disabled respondents, the association was not significant. SP is effective in decreasing depressive symptom severity in disabled older populations. Diversified activities and targeted interventions should be applied to specified older disabled populations to prevent depression.

## KEY POINTS

- Correlations between depressives symptom severity and social participation (SP) among disable older adults were measured in a Chinese context.
- Pooled Ordinary Least Square regression were used to explore the impact of SP on depressives symptom severity in older populations with disabilities.
- Social participation, including recreational activities and interacting with friends, was associated with decreased depressives symptom severity in disabled middle-aged and older adults.
- The impact of SP on depressives symptom severity was not significant for older adults with brain disabilities.

## Introduction

Increasing life expectancy is driving the rapid aging of the world’s population, especially in developing countries (Seals et al., 2016). The World Health Organization defines “healthy aging” as the development and maintenance of functional capacities that enable older people to achieve well-being (Sanchez-Sanchez et al., 2020). There is evidence that disability takes away from the years we gain and that progress in life expectancy is eroded by disability, which further hinders healthy aging (Kim et al., 2021). Disabilities in older adults can lead to cognitive decline, decreased independence, and a lower quality of life (LaMonica et al., 2019). The World Report on Disability has revealed that more than one billion people worldwide – about 15% of the total global population – live with some kind of disability. The number of people with disability will continue to rise as populations age, alongside the aggravation of chronic health conditions (Cieza et al., 2018). Therefore, disability may be one of the most important factors affecting healthy aging at present. China is one of the world’s fastest aging countries and has the largest aging population in the world (Cheng & Yan, 2021). It is important to give high priority to disability and develop timely interventions to improve healthy aging, reduce disability morbidity and improve quality of life (Osth et al., 2019).

The mental health of the disabled population is of great concern. Because of their shrinking social networks, older adults with disabilities tend to have low self-identification and a high sense of loneliness or meaninglessness, placing them at greater risk for cognitive impairment, suicide, and death (Li et al., 2020). Moreover, it has been confirmed that depression and disability are closely related, with depression being one of the leading causes of disability worldwide (Murphy et al., 2016), and that disability directly and significantly predicts depression, resulting in consequences such as discrimination, stigma, and lowered self-esteem for individuals with disabilities, further affecting their quality of life and being detrimental to healthy aging (Trani et al., 2020).

Factors that can decrease depressive symptom severity in individuals with disabilities need to be identified and social participation (SP) may be one of the key strategies. The literature suggests that more social support can be obtained through SP, which reduces the risk of experiencing psychological stress (Zhou et al., 2020). Integration into social networks can reduce stress during adverse life events and improve coping with disability later in life (Ellwardt et al., 2020). People who participate in social activities, including social interactions and leisure activities, have better mental health and higher health literacy (Iwasa & Yoshida, 2018). In addition, mental health can be influenced by SP through improvements in subjective well-being (Cheng & Yan, 2021). Nevertheless, whether SP is effective among the disabled older population has not been well explored. A previous review indicated that social relationships play an important role in mental health and well-being in persons with disabilities, although findings are less consistent than in general populations and strength of associations vary between constructs (Tough et al., 2017). However, most of the studies concentrated more on social support from families, relatives/friends, or communities, rather than interactions initiated by disabled older adults themselves proactively. In other words, if those disabled could take part in social activities spontaneously, their depressive symptom severity may be reduced. However, the evidence is scarce. Furthermore, different SP types may generate different impacts on depression (Wang et al., 2019). The depressive symptom severity may also vary under different disability types and disability levels. For example, in a cross-sectional study, among adults with disabilities in the United States, those with both cognitive and mobility disabilities most frequently reported mental distress (55.6%) (Cree et al., 2020). The research related to depressive symptom severity impacted by different kinds of SP among different disability types is far from adequate.

Therefore, we propose a research hypothesis that SP can decrease the depressive symptom severity of disabled older populations, and that the effect varies among different SP types (recreational activities and interacting with friends), disability types (physical disability, brain damage/intellectual disability, and facial featured disability), and disability levels (none, mild and severe). The aim of this study was to explore the associations between SP and depressive symptom severity among middle-aged and older populations with disability, as well as the associations based on different disability and SP types.

## Methods

### Participants

**Figure 1.**
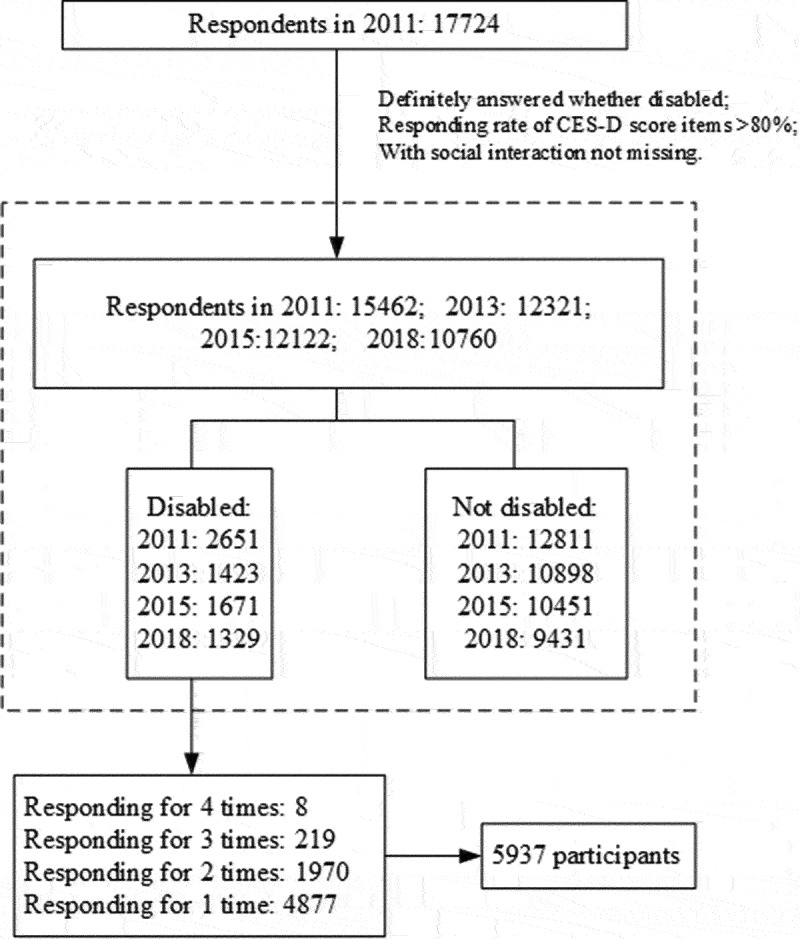
Flowchart of sample selection.

Data used for this study were from the China Health and Retirement Longitudinal Study (CHARLS), conducted in 2011, 2013, 2015, and 2018 by the National School of Development of Peking University. The data and sampling details can be found on its official website (http://charls.pku.edu.cn/) and previous studies (Zhao et al., 2014). The baseline sample included 17,224 adults aged ≥45 years. Sample selection is detailed in Figure 1.

## Measures

### Dependent variable

The respondents’ depressive symptom severity was measured by the 10-item form of the Center for Epidemiological Studies Depression (CES-D) scale, with a response scale ranging from 0 (rarely or never) to 3 (most or all the time). The summed scores ranged from 0 to 30, with higher scores indicating negative feelings and a higher depressive symptom severity (Diego et al., 2001).

### Independent variable

The primary independent variable in this study was SP. The SP in the CHARLS included interacting with friends; playing mah-jongg, chess, cards, or going to other community clubs; going to a sport, social, or other club; taking part in community-related organizations; undertaking voluntary or charitable work; providing help to relatives, friends, or neighbors who do not live with the respondent for free; and using the internet. For disabled respondents, many SP were limited or difficult for them (Table 1); thus, only interacting with friends and recreational activities (playing mah-jongg, chess, cards, or going to other community clubs) were analyzed as subgroups.

**Table 1.** Description of the disabled sample*.

	2011 (*n* = 2651)	2013 (*n* = 1423)	2015 (*n* = 1671)	2018 (*n* = 1329)
Age	62.90 ± 10.48	63.71 ± 9.23	64.61 ± 9.01	65.99 ± 8.32
Gender				
Male	1376 (51.90%)	728 (51.16%)	793 (47.46%)	632 (47.55%)
Female	1275 (48.10%)	695 (48.84%)	878 (52.54%)	697 (52.45%)
Educational level^a^				
Illiteracy	2471 (93.21%)	1308 (91.92%)	1549 (92.70%)	1211 (91.12%)
Secondary education1	151 (5.70%)	102 (7.17%)	106 (6.34%)	110 (8.28%)
Higher education	29 (1.09%)	13 (0.91%)	16 (0.96%)	8 (0.60%)
Marital status				
Not married	520 (19.62%)	250 (17.59%)	326 (19.52%)	253 (19.04%)
Married	2131 (80.38%)	1171 (82.41%)	1344 (80.48%)	1076 (80.96%)
Hukou status^b^				
Agriculture	2205 (83.21%)	1138 (80.31%)	1256 (82.15%)	1113 (83.87%)
Non-agriculture	432 (16.30%)	263 (18.56%)	246 (16.09%)	214 (16.13%)
Unified residency	13 (0.49%)	16 (1.13%)	27 (1.77%)	0 (0.00%)
Public health insurance coverage^c^				
No	207 (7.84%)	65 (4.62%)	161 (9.65%)	55 (4.14%)
Yes	2432 (92.16%)	1343 (95.38%)	1508 (90.35%)	1274 (95.86%)
Household per capita consumption^d^	6287.82 ± 8142.38	10435.95 ± 11522.04	13582.94 ± 26089.64	20310.61 ± 43798.57
Employment status^e^				
No	1092 (41.39%)	591 (41.91%)	694 (41.78%)	601 (45.22%)
Yes	1546 (58.61%)	819 (58.09%)	967 (58.22%)	728 (54.78%)
Smoking status				
Never	1447 (54.60%)	760 (69.41%)	894 (53.57%)	755 (56.81%)
Quit	324 (12.23%)	143 (13.06%)	327 (19.59%)	240 (18.06%)
Still	879 (33.17%)	192 (17.53%)	448 (26.84%)	334 (25.13%)
Alcohol intake				
No	1801 (67.94%)	946 (66.67%)	1166 (69.95%)	924 (69.53%)
Yes	850 (32.06%)	473 (33.33%)	501 (30.05%)	405 (30.47%)
Residence^f^				
Rural	1813 (68.39%)	916 (64.37%)	1150 (68.82%)	905 (68.10%)
Urban	838 (31.61%)	507 (35.63%)	521 (31.18%)	424 (31.90%)
ADL-6	0.82 ± 1.45	0.80 ± 1.37	1.03 ± 1.53	1.03 ± 1.52
Chronic conditions^g^	1.82 ± 1.54	2.22 ± 1.73	2.74 ± 1.87	3.44 ± 2.20
Self-rated health^h^				
Unhealthy	2245(84.69%)	1232(86.58%)	1455(87.07%)	1189(89.47%)
Healthy	406(15.31%)	191(13.42%)	216(12.93%)	140(10.53%)
Disability level				
None	1736(65.91%)	916(64.37%)	937(56.07%)	638(55.09%)
Mild	560(21.26%)	334(23.47%)	464(27.77%)	333(28.76%)
Severe	338(12.83%)	173(12.16%)	270(16.16%)	187(16.15%)
SP types				
Interacting with friends	813 (30.67%)	526 (36.96%)	563 (33.69%)	395 (29.72%)
Take part in a community-related organization	30 (1.13%)	26 (1.83%)	38 (2.27%)	23 (1.73%)
Recreational activities	366 (13.81%)	235 (16.51%)	274 (16.40%)	184 (13.84%)
Go to a sport, social or other clubs	89 (3.36%)	97 (6.82%)	106 (6.34%)	59 (4.44%)
Voluntary or charitable work	10 (0.38%)	15 (1.05%)	30 (1.80%)	12 (0.90%)
Use the internet	20 (0.75%)	26 (1.83%)	40 (2.39%)	68 (5.12%)
Disability types				
Physical disability	422 (15.92%)	197 (13.84%)	255 (15.26%)	197 (14.82%)
Brain damage/intellectual disability	212 (8.00%)	208 (14.62%)	178 (10.65%)	209 (15.73%)
Facial feature disabled	1707 (64.39%)	865 (60.79%)	1072 (64.15%)	772 (58.09%)
Physical disability & brain damage/intellectual disability	34 (1.28%)	17 (1.19%)	17 (1.02%)	13 (0.98%)
Physical disability & facial feature disabled	145 (5.47%)	57 (4.01%)	54 (3.23%)	50 (3.76%)
Brain damage/intellectual disability & facial feature disabled	104 (3.92%)	69 (4.85%)	77 (4.61%)	74 (5.57%)
Physical disability & brain damage/intellectual disability & facial feature disabled	27 (1.02%)	10 (0.70%)	18 (1.08%)	14 (1.05%)

Note: Mean±standard deviation was used to describe continuous variable and number (constituent ratio [%]) was used to describe categorical variable.

^a^Education level is a simplified version of 1997 International Standard Classification of Education codes.

^b^Hukou status indicates the respondent’s hukou place and is a special identifier in China, which affects many aspects of life in China such as buying a house, children’s school, and other welfare.

^c^Covered by public health insurance indicates that the respondent is covered by at least one type of public health insurance plan, including Urban Employee Medical Insurance, Urban Resident Medical Insurance, New Cooperative Medical Insurance, Urban and Rural Resident Medical Insurance, Government Medical Insurance, Medical Aid or other government insurance plan.

^d^Household per capita consumption is calculated by taking total household consumption divided by the number of people in the household. The amount of total household consumption as aggregated from all consumption activities: food consumption in last week, non-food in the past 30 days, and other non-food consumption in the past year.

^e^Employment status indicates whether the respondent engaged in any work in the past year, including agricultural work, non-agricultural employed work, non-agricultural self-employment work, or non-agricultural family business work per the labor force status of each wave respectively.

^f^Residence indicates the household living region and is defined by National Bureau of Statistics of the People’s Republic of China.

^g^Chronic conditions indicates the number of chronic diseases that the respondent is suffering from, including hypertension, diabetes, dyslipidemia, heart disease, stroke, cancer, chronic lung disease, digestive disease, liver disease, kidney disease, and arthritis.

^h^Respondents were classified as “unhealthy” if they rated their health status as “not healthy” or “poor,” and as “healthy” if they rated their health status as “common,” “healthy” or “very healthy.”

### Covariates

Based on previous research, the following participant sociodemographic characteristics were selected as control variables: age, gender, marital status, rural/urban residence, educational level, Hukou status, household per capita consumption, public health insurance coverage, employment status, disability level (6 items of activities of daily living [ADL-6]), self-rated health, chronic conditions, and health behaviors (alcohol intake and smoking status) (Chen et al., 2012; Feng et al., 2021; Ji et al., 2021). The definitions and categorizing details are presented in Table 1 and footnotes.

## Statistical analysis

Smooth curve fitting for mean CES-D scores across different survey waves were generated based on general additive models (GAMs), while adjusting for all covariates, to compare the level and trajectory of CES-D scores between disabled respondents and those not disabled. In CHARLS, most disabled participants responded in only one wave (details for responding times were shown in Figure 1). Therefore, cross-sectional design was used, and analyzed data were compiled by data across different survey waves. Pooled Ordinary Least Square (OLS) regression models were applied to explore associations between CES-D scores and SP among disabled respondents. We included individual and survey wave as fixed effects, in order to capture the impact of any unobserved individual and time trend. Standard errors are clustered at the individual level to allow for autocorrelation of observations (Ferrari & Salustri, 2020). Magnitude of the effects were measured by effect sizes and presented by coefficients (β) (Cooksey-Stowers et al., 2017).

The analysis strategies were as follows:

1. To identify associations between CES-D scores and SP, we used three models: the crude model without adjustment for any covariates; a model adjusted for age and gender; a model adjusted for all the selected covariates.
2. We explored these associations across different SP types, including interacting with friends and recreational activities, and repeated the assessment with the three models mentioned above.
3. We identified associations between CES-D scores and SP among respondents with different disability types and disability levels. Considering the sample size (Table 1), we mainly concentrated the results of the single-type disability (physical disability, brain damage/intellectual disability, and facial feature disabilities such as vision problem, hearing problem, and speech impediment). Disability level was classified as none, mild, and severe based on ADL-6 (controlling urination and defecation, dressing, bathing/shower, eating, getting in/out bed, using the toilet). Participants were considered to have a mild level of disability if they answered “I am unable to do it by myself” or “I have any difficulty with an activity” for one or two items, and a severe level for more than two items (Carmona-Torres et al., 2019; Feng et al., 2021).
4. We identified associations between CES-D scores and different SP types (interacting with friends and recreational activities) across the different disability types with all the covariates adjusted.
5. We explored all the associations above between different genders and residences (rural or urban).

In addition, SP benefits may differ depending on the employment status of the participants (Araten-Bergman et al., 2015; Martins, 2015). Therefore, subgroup analysis of employment status was further conducted. When analyzing disability type, only single-type disabled participants were enrolled.

We conducted validation analysis three ways. First, we selected disabled participants only responding in one survey wave to explore the association between CES-D scores and SP. Second, we performed multiple imputation (MI) to avoid statistical test performance reduction and bias due to the direct exclusion of missing values, based on ten replications and a chained equation approach (White et al., 2011). Third, we repeated OLS regression using data of three waves (2011-2015).

Mean ± standard deviation and number (percentages) were used for descriptions of the respondents’ characteristics. The P-values were 2-sided, and an alpha level of 0.05 was used to define statistical significance. Data were analyzed using Stata (version 15) and R version 3.6.3 (R Foundation for Statistical Computing, Vienna, Austria).

## Results

**Figure 2.**
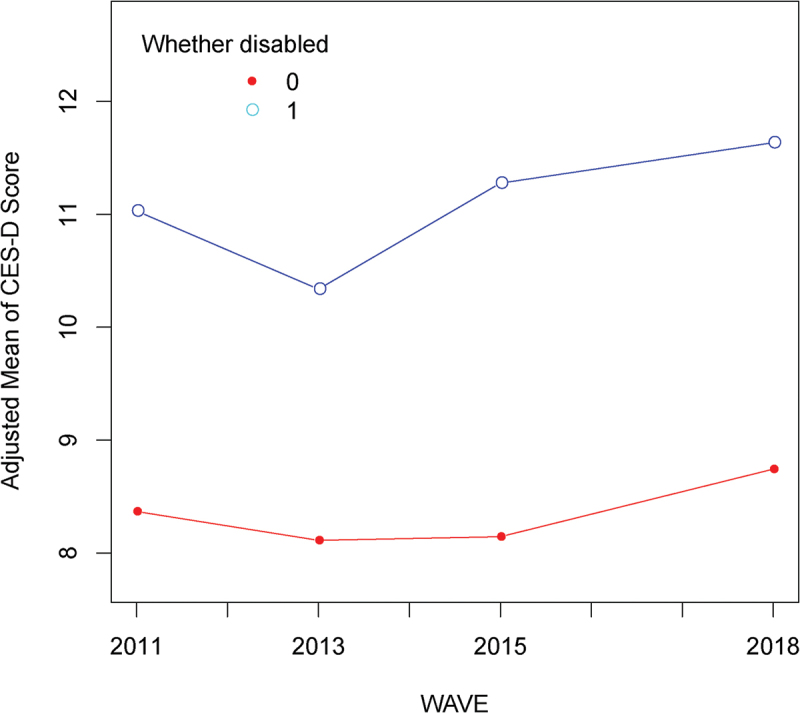
Smooth curve fitting for CES-D scores in disabled and nondisabled respondents across the 4 survey waves. 0: non-disabled; 1: disabled.

The description of the whole sample is shown in Supplementary Table S1. We compared mean CES-D scores between the disabled group and the nondisabled group (Supplementary Table S2), and a higher mean CES-D score was observed in the disabled group than in the nondisabled group in each wave. The mean CES-D scores for the whole sample ranged from 7.91 in wave 2 to 8.80 in wave 4, and from 10.15 in wave 2 to 11.57 in wave 4 for the disabled group samples. Smooth curve fitting for the trajectory of CES-D scores for the disabled and nondisabled respondents (Figure 2) also revealed a higher depression risk in the disabled group, with all the covariates adjusted. Then, disabled respondents (*n* = 5937) were selected for all follow-up analyses.

Table 1 shows the characteristics of the disabled participants across 4 waves. The mean ages were 62.90 and 65.99 years old in 2011 and 2018, respectively. Half of the respondents were males. Most were married and had an agriculture Hukou. The prevalence of employment showed a slight decreasing trend from 58.61% to 54.78%. Two-thirds seldom drank. The prevalence of smoking was under 35%.

We explored the effects of SP, as well as different SP types, on CES-D scores in those with disabilities based on pooled OLS models (Table 2). The OLS models showed a significant negative relationship between SP and CES-D scores among the disabled population, whether controlling for no covariates (unadjusted), controlling for age and gender only (adjusted 1) and controlling for all the covariates (adjusted 2). With all the covariates adjusted, disabled respondents with SP had lower CES-D scores (β= −0.96; 95% CI: −1.31 to −0.62; *P* < .001), and similar associations with CES-D scores were apparent with different SP types (recreational activities: β= −1.30; 95% CI: −1.78 to −0.81; *P* < .001; interacting with friends: β= −0.82; 95% CI: −1.19 to −0.46; *P* < .001).

**Table 2.** Associations between CES-D scores and SP based on OLS regressions (*n* = 5937).

	Unadjusted		Adjusted 1*		Adjusted 2^#^	
	β (95%CI)	P value	β (95%CI)	P value	β (95%CI)	P value
SP	−1.63(−1.96,-1.31)	<0.001	−1.62(−1.94,-1.30)	<0.001	−0.96(−1.31,-0.62)	<0.001
Types of SP						
Recreational activities	−2.37(−2.82,-1.92)	<0.001	−2.02(−2.47,-1.57)	<0.001	−1.30(−1.78,-0.81)	<0.001
Interacting with friends	−1.05(−1.39,-0.70)	<0.001	−1.17(−1.51,-0.83)	<0.001	−0.82(−1.19,-0.46)	<0.001

Note: SP, social participation; OLS, Ordinary Least Square; CI, confidence interval.

*Adjust 1: Adjusting for age and gender.

^#^Adjust 2: Adjusting for age, gender, marital status, rural/urban residence, educational level, Hukou status, household per capita consumption, public health insurance coverage, employment status, ADL-6, self-rated health, chronic conditions, alcohol intake and smoking status.

We subsequently used OLS models across different types of disability after controlling for all the covariates (Table 3). SP was negatively related to CES-D scores for respondents with physical disabilities (β=-1.55; 95% CI: −2.50 to −0.60; *P* = .001) and facial feature disabilities (β=-0.83; 95% CI: −1.25 to −0.40; *P* < .001), and this association was also significant among respondents with different disability levels. Moreover, participation in recreational activities and interacting with friends both had a significant impact on CES-D scores for the two disability types and three disability levels, except for the impact of recreational activities on severely disabled respondents (*P* = .091). For respondents with brain damage/intellectual disability, the impact of SP was not significant.

**Table 3.** Associations between SP and CES-D scores among the disabled population with different disability types based on OLS models*.

	SP		Types of SP			
			Recreational activities		Interacting with friends	
	β (95%CI)	P value	β (95%CI)	P value	β (95%CI)	P value
**Disability type^a^**						
Physical disability	−1.55(−2.50,-0.60)	0.001	−1.61(−2.91,-0.31)	0.015	−1.22(−2.22,-0.21)	0.018
Brain damage/intellectual disability	−0.82(−1.85,0.20)	0.115	−0.55(−1.94,0.85)	0.441	−0.93(−2.05,0.19)	0.102
Facial feature disabled	−0.83(−1.25,-0.40)	<0.001	−1.37(−1.97,-0.77)	<0.001	−0.64(−1.08,-0.20)	0.005
**Disability level^b^**						
None	−0.64(−1.06,-0.22)	0.003	−1.02(−1.58,-0.46)	<0.001	−0.62(−1.06,-0.18)	0.005
Mild	−1.32(−2.03,-0.61)	<0.001	−2.05(−3.11,-1.00)	<0.001	−0.94(−1.70,-0.19)	0.014
Severe	−1.77(−2.83,-0.72)	0.001	−1.63(−3.53,0.26)	0.091	−1.47(−2.59,-0.35)	0.010

Note: SP, social participation; OLS, Ordinary Least Square; CI, confidence interval.

^a^Adjusted factors include age, gender, marital status, rural/urban residence, educational level, Hukou status, household per capita consumption, public health insurance coverage, employment status, ADL-6, self-rated health, chronic conditions, alcohol intake and smoking status.

^b^Adjusted factors include all the covariates except for ADL-6.

Subgroup analysis revealed negative associations between SP and CES-D scores for the subgroup of males (β= −0.11; 95% CI: −1.64 to −0.57; *P* < .001), females (β= −1.39; 95% CI: −1.96 to −0.81; *P* < .001), rural respondents (β= −0.88; 95% CI: −1.37 to −0.40; *P* < .001) and urban respondents (β= −2.04; 95% CI: −2.71 to −1.36; *P* < .001), employed respondents (β= −0.71; 95% CI: −1.16 to −0.27; *P* = .002) and those not employed (β= −1.34; 95% CI: −1.87 to −0.80; *P* < .001). These associations remained significant and consistent when examining recreational activities and interacting with friends. Nevertheless, when analyzing different disability types, we found that the impact of SP on CES-D scores was not significant among those with brain disabilities in all subgroups with the exception of females and those not employed, which may be caused by small sample size. The details are shown in Table 4.

**Table 4.** Subgroup analyses based on OLS regressions*.

	Gender^a^		Residence^b^		Employemnt status^c^	
	Male	Female	Rural	Urban	Employed	Not employed
SP	−0.57(−1.05,-0.10)**	−1.32(−1.80,0.83)****	−0.83(−1.25,0.41)****	−1.25(−1.85,0.65)****	−0.71(−1.16,-0.27)***	−1.34(−1.87,-0.80)****
Types of SP						
Recreational activities	−0.88(−1.48,-0.27)***	−1.94(−2.72,1.16)****	−1.07(−1.70,-0.45)***	−1.64(−2.39,0.88)****	−0.98(−1.60,-0.36)***	−1.71(−2.47,-0.94)****
Interacting with friends	−0.57(−1.09,-0.06)**	−1.01(−1.51,0.50)****	−0.85(−1.29,0.41)****	−0.75(−1.38,-0.12)**	−0.90(−1.37,-0.43)****	−0.72(−1.28,-0.15)**
Types of disability						
Physical disability	−1.45(−2.67,-0.22)**	−2.08(−3.61,-0.55)***	−1.68(−2.86,-0.50)***	−1.54(−3.19,0.11)*	−1.67(−2.93,-0.41)***	−1.83(−3.31,-0.36)**
Brain damage/intellectual disability	0.21(−1.22,1.64)	−1.65(−3.14,-0.17)**	−0.75(−2.07,0.58)	−1.06(−2.72,0.61)	0.65(−0.78,2.07)	−2.72(−4.24,-1.21)****
Facial feature disabled	−0.48(−1.09,0.13)	−1.08(−1.66,0.49)****	−0.62(−1.13,-0.11)**	−1.29(−2.05,-0.54)***	−0.62(−1.15,-0.09)**	−1.10(−1.79,-0.40)***

Note: SP, social participation; OLS, Ordinary Least Square; CI, confidence interval. *P < .01; **P < .05; ***P < .01; ****P < .001.

^a^Adjusted factors include all the covariates except for gender.

^b^Adjusted factors include all the covariates except for residence.

^c^Adjusted factors include all the covariates except for employment status.

Three sensitivity analyses were conducted to validate the association between SP and depression among the disabled older adults. The results revealed consistent conclusions, with consistent direction and magnitude of the effect (Supplementary Table S4).

## Discussion

The present study indicated that the severity of depressive symptoms was chronically higher in the disabled group than in the nondisabled group among the middle-aged and older population. For the disabled respondents, SP was found to have a significant effect on reducing depressive symptom severity, including interaction with friends and recreational activities. Consistent results were obtained when analyzed separately across disability types, with the exception of those with brain disabilities, whose depressive symptom severity was not significantly affected by SP.

It has been documented that people with disabilities have a significantly higher prevalence of mental illness and face an increased risk of co-occurring psychological distress compared to the general population (Houston et al., 2016). They may have negative states such as low self-esteem and stigma (Trani et al., 2020), due to their lack of independent control and low self-esteem when faced with the fact of their disability (Gómez Díaz & Jiménez García, 2018). Therefore, improving the mental health of disabled older adults is essential and urgent for the process of healthy aging.

SP is an effective way to decrease depressive symptom severity among older populations with disabilities. Previous studies have shown that the needs of older adults with disabilities in terms of SP are rarely met; these activities include leisure, other community life activities and interpersonal relationships, and some daily activities, including fitness and mobility exercises (Turcotte et al., 2015). At the same time, reduced SP is likely to compound the decreased quality of life of people with physical disabilities who are lonely and depressed (Steptoe & Gessa, 2021). In contrast, SP can show improvements with regard to the onset of disability and can have a positive effect on the mental state of the disabled population by providing them with a sense of meaning in life through enjoyable interactions with others in society (Hikichi et al., 2015). Meanwhile, SP in this study, such as interaction with friends and recreational activities, mainly refers to proactive participation by respondents. These activities are also associated with stimulated intellect and, consequently, maintained cognitive function; more sufficient social network support and decreased risk of social isolation; loneliness; and depression among general older adults (Iizuka et al., 2019). This study further proved their effectiveness among disabled older adults. Therefore, on one hand, families and communities should encourage the disabled older population to actively take part in social activities. On the other hand, we should encourage the community to organize more activities and design individualized social events for people with disabilities to make them feel socially supported and thus promote mental health.

For people with brain disabilities, the effect of SP on depressive symptom severity was limited. This could be because individuals with intellectual disability may have neurodevelopmental deficits characterized by limitations in intellectual functioning and adaptive behavior which complicates intervention. In addition, the depressive symptom severity among these populations is higher than the general population (Whitney et al., 2019), which indicates that it may be more difficult to prevent depression. Appropriate and ongoing education of people with brain disabilities and their caregivers is needed to enable them to live healthy lives in the areas of physical, social, and cognitive activity through other means (Reppermund & Trollor, 2016). Supporting and assisting people with brain disabilities in decision-making by making their will and preferences central to their decisions is a way to increase their control over their lives and positively impact self-identity, mental health, and quality of life (Douglas & Bigby, 2020).

## Limitations

First, this study suffers from inherent flaws of retrospective studies, such as recall bias, which indicates that the relationship between SP and depression requires a definitive validation in prospective studies. Second, this study supported the association between SP and depressive symptom severity; however, the mechanisms need to be further explored. Third, though different types of disability and disability levels were assessed, a more accurate disability type – such as the part of body affected by disability – could not be obtained through the database. Fourth, the sample size of participants interviewed across all 4 survey waves was limited. Thus, longitudinal design or cohort analysis was not appropriate for this study and causal effects could not be assessed. However, we conducted several sensitivity and subgroup analyses, and the results were robust. In light of these limitations, subsequent prospective studies are needed to examine the most effective measures associated with SP to prevent depression of disabled older populations.

## Conclusions

The disabled population is more vulnerable among middle-aged and older individuals, with a higher risk of depression than those who are not disabled. SP is an effective way to reduce depressive symptom severity in the disabled older population. They should be encouraged to take part in social activities more proactively and the community should organize more and diversified activities for the disabled people to make them feel socially supported. In addition, for older adults with brain damage/intellectual disability, the effect of SP should be strengthened by more targeted measures, intelligent devices, and family and social support.

## Supplementary Material

Supplemental Material

## Data Availability

Data are available in a public, open access repository. All data can be accessed and downloaded at the CHARLS home page (http://charls.pku.edu.cn/en).
