# Supplementary material for: Social Participation: A Strategy to Manage Depression in Disabled Populations
Source: J Aging Soc Policy. 2023 Sep 12;37(2):324–40. doi: 10.1080/08959420.2023.2255492 (PMC11875427; doi:10.1080/08959420.2023.2255492)
Supplement: Supplemental Material [file WASP_A_2255492_SM4534.docx]

**Social participation: Strategy to manage depression in disabled populations**

**Supplementary Tables**

**Table S1.** Description of the whole sample.

**Table S2.** Distribution in CES-D scores in the disabled and nondisabled groups (mean±SD）

**Table S3.** Sensitivity analysis based on OLS regression

**Table S1.** Description of the whole sample.

|  | **2011** | **2013** | **2015** | **2018** |
| --- | --- | --- | --- | --- |
| Age | 59.05 ± 9.66 | 60.56 ± 9.04 | 62.15 ± 8.82 | 64.21 ± 8.21 |
| Gender |  |  |  |  |
| Male | 7380 (47.73%) | 5859 (47.55%) | 5776 (47.65%) | 5049 (46.92%) |
| Female | 8082 (52.27%) | 6462 (52.45%) | 6346 (52.35%) | 5711 (53.08%) |
| Educational level |  |  |  |  |
| Illiteracy | 13518 (87.43%) | 10872 (88.24%) | 10733 (88.54%) | 9495 (88.24%) |
| Secondary education1 | 1595 (10.32%) | 1237 (10.04%) | 1181 (9.74%) | 1114 (10.35%) |
| Higher education | 348 (2.25%) | 212 (1.72%) | 208 (1.72%) | 151(1.40%) |
| Marital status |  |  |  |  |
| Not married | 1979 (12.80%) | 1600 (12.99%) | 1763 (14.55%) | 1738 (16.15%) |
| Married | 13483 (87.20%) | 10716(87.01%) | 10358(85.45%) | 9022 (83.85%) |
| Hukou status |  |  |  |  |
| Agriculture | 11947 (77.29%) | 9612 (78.18%) | 9099 (79.47%) | 8685 (80.87%) |
| Non-agriculture | 3414 (22.09%) | 2547 (20.72%) | 2094 (18.29%) | 2053 (19.12%) |
| Unified residency | 96 (0.62%) | 135 (1.10%) | 256 (2.24%) | 1 (0.01%) |
| Public health insurance |  |  |  |  |
| No | 1190 (7.73%) | 470 (3.84%) | 1090 (9.00%) | 385 (3.58%) |
| Yes | 14207 (92.27%) | 11764 (96.16%) | 11026(91.00%) | 10375 (96.42%) |
| Household per capita consumption | 7826.85±21210.32 | 10626.08±13893.39 | 13675.06±21802.96 | 21560.73±42509.18 |
| Employment status |  |  |  |  |
| No | 5016 (32.58%) | 3842 (31.38%) | 4125 (34.22%) | 4097 (38.08%) |
| Yes | 10378 (67.42%) | 8401 (68.62%) | 7929 (65.78%) | 6662 (61.92%) |
| Smoking status |  |  |  |  |
| Never | 9353 (60.50%) | 7031 (74.83%) | 6702 (55.32%) | 6256 (58.14%) |
| Quit | 1378 (8.91%) | 834 (8.88%) | 2068 (17.07%) | 1664 (15.46%) |
| Still | 4728 (30.58%) | 1531 (16.29%) | 3345 (27.61%) | 2840 (26.39%) |
| Alcohol intake |  |  |  |  |
| No | 10423 (67.41%) | 8163 (66.32%) | 8039 (66.35%) | 7268 (67.55%) |
| Yes | 5039(32.59%) | 4146 (33.68%) | 4077 (33.65%) | 3492 (32.45%) |
| Residence |  |  |  |  |
| Rural | 9209 (59.56%) | 7656 (62.14%) | 7674 (63.31%) | 6790 (63.10%) |
| Urban | 6253 (40.44%) | 4665 (37.86%) | 4448 (36.69%) | 3970 (36.90%) |
| ADL-6 | 0.34±0.97 | 0.32±0.87 | 0.42±1.01 | 0.42±1.03 |
| Chronic condition | 1.44±1.41 | 1.65±1.51 | 2.18±1.74 | 2.66±1.97 |
| Social activities |  |  |  |  |
| No | 7751(50.15%) | 5300(43.02%) | 5751(47.44%) | 5306(49.31%) |
| Yes | 7704(49.85%) | 7021(56.98%) | 6371(52.56%) | 5454(50.69%) |
| Self-rated health |  |  |  |  |
| Unhealth | 11527(74.55%) | 9290(75.41%) | 9351(77.14%) | 8375(77.86%) |
| Health | 3935(25.45%) | 3029(24.59%) | 2771(22.86%) | 2381(22.14%) |

**Table S2.** **Distribution in CES-D scores in the disabled and nondisabled groups (mean±SD）**

|  | 2011 | 2013 | 2015 | 2018 |
| --- | --- | --- | --- | --- |
| Overall | 8.43 ± 6.36 | 7.91 ± 5.81 | 8.25 ± 6.48 | 8.80 ± 6.59 |
| Disabled | 11.07 ± 7.00 | 10.15 ± 6.42 | 11.11 ± 7.18 | 11.57 ± 7.23 |
| Not disabled | 7.89 ± 6.09 | 7.62 ± 5.66 | 7.80 ± 6.25 | 8.42 ± 6.40 |

SD, standard deviation.

**Table S3. Sensitivity analysis based on OLS regression***

|  | β(95%CI) | P value |
| --- | --- | --- |
| Participants only responding in one survey wave (n=4877) | -1.05 (-1.34 to -0.76) | <0.001 |
| Multiple imputation (n=5937)^#^ | -0.88 (-1.29 to -0.47) | <0.001 |
| Participants in 2011-2015 (n=5067) | -0.90 (-1.28 to -0.52) | <0.001 |

OLS, Ordinary Least Square; CI, confidence interval.

^*^ The OLS regressions were applied to explore the association between social interactions and CES-D scores. Adjusted factors include age, gender, marital status, rural/urban residence, educational level, Hukou status, household per capita consumption, public health insurance coverage, employment status, ADL-6, self-rated health, chronic conditions, alcohol intake and smoking status.

^#^ The imputing method was from the posterior predictive distribution (ppd). Ten sets of data were generated, and the regression coefficients were pooled.
